# Calcium-dependent conformational transitions and ligand binding regulation of C-reactive protein: a comprehensive review

**DOI:** 10.3389/fimmu.2026.1963529

**Published:** 2026-10-12

**Authors:** Bin Cheng, Zhi-Xin Yao, Guo-Qiang Wu

**Affiliations:** 1 School of Life Sciences and Engineering, Lanzhou University of Technology, Lanzhou, China; 2 School of Materials Science and Engineering, Lanzhou University of Technology, Lanzhou, China

**Keywords:** calcium ion, complement, C-reactive protein, inflammation, ligand, pentamer

## Abstract

C-reactive protein (CRP) is a crucial biomarker of inflammation and a primary pattern recognition molecule of the innate immune system. It is predominantly synthesized by hepatocytes and subsequently released into the circulation. The concentration of CRP in plasma can increase significantly, sometimes by up to a thousand-fold, in the presence of infection, inflammation, or tissue damage, making it a widely used clinical indicator of inflammatory status. The distinctive structure of pentameric CRP (pCRP), coupled with the specific functions of its recognition face (B-face) and effector face (A-face) as well as its calcium-dependent ligand recognition, dictates its structural and functional characteristics. This review presents a thorough analysis of the structure and functional surfaces of CRP, focusing on the dual regulatory role of calcium ions (Ca^2+^) in maintaining structural stability and facilitating ligand recognition. It further examines the historical and mechanistic basis of CRP conformational conversion, emphasizing the distinction between Ca^2+^ depletion-induced destabilization and physiologically relevant membrane-associated remodeling that can occur in the continued presence of Ca^2+^. The present review explores the interaction mechanisms of CRP with its ligands, as well as the influence of the microenvironment on CRP’s conformational dynamics and functional diversity. This review could provide new prospects for designing targeted drugs according to the structural characteristics of CRP.

## 1 Introduction

C-reactive protein (CRP) is a classic representative of acute-phase reactants and a member of the short pentraxin family (1, 2). Its plasma levels can rapidly increase by hundreds to thousands of times within hours following infection, tissue injury, or inflammation, making it a valuable serum biomarker for assessing inflammatory states and tissue damage (3, 4). Beyond its role as a biomarker, CRP acts as a pattern recognition molecule in the innate immune system, identifying pathogen surfaces and endogenous ligands from apoptotic or damaged cells (5), particularly molecular patterns containing phosphocholine (PC). This recognition process further activates the classical complement pathway, promoting opsonic phagocytosis, pathogen clearance, and early host immune defense (6–8).

CRP is a pentameric protein composed of five identical subunits assembled through noncovalent interactions. The symmetrical structure of pentameric CRP (pCRP), together with its distinct bifunctional surfaces and calcium-dependent ligand recognition, provides the structural basis for its immunological functions (9–11). The recognition face (B-face), responsible for ligand recognition, contains two essential calcium ion (Ca^2+^)-binding sites for PC recognition, whereas the effector face (A-face) functions as the effector surface by interacting with downstream molecules such as complement C1q and immune cell receptors. This spatial coupling of target recognition and effector recruitment enables precise regulation of innate immune responses by integrating recognition and elimination processes (12, 13).

Recent advances in structural biology and high-resolution techniques have enhanced our comprehension of CRP’s structural complexity, conformational dynamics, and interactions with ligands and receptor complexes (13). This review aims to analyze the molecular architecture of the pCRP and its unique functional properties, investigate the crucial role of Ca^2+^ in maintaining protein conformational stability and regulating ligand recognition, and systematically elucidate the specific binding mechanisms of CRP with ligands and their structural basis. These insights provide a foundation for further elucidating CRP’s functional mechanisms in physiological and pathological processes and for developing targeted intervention strategies centered on CRP (**Figure 1**).

**Figure 1:**
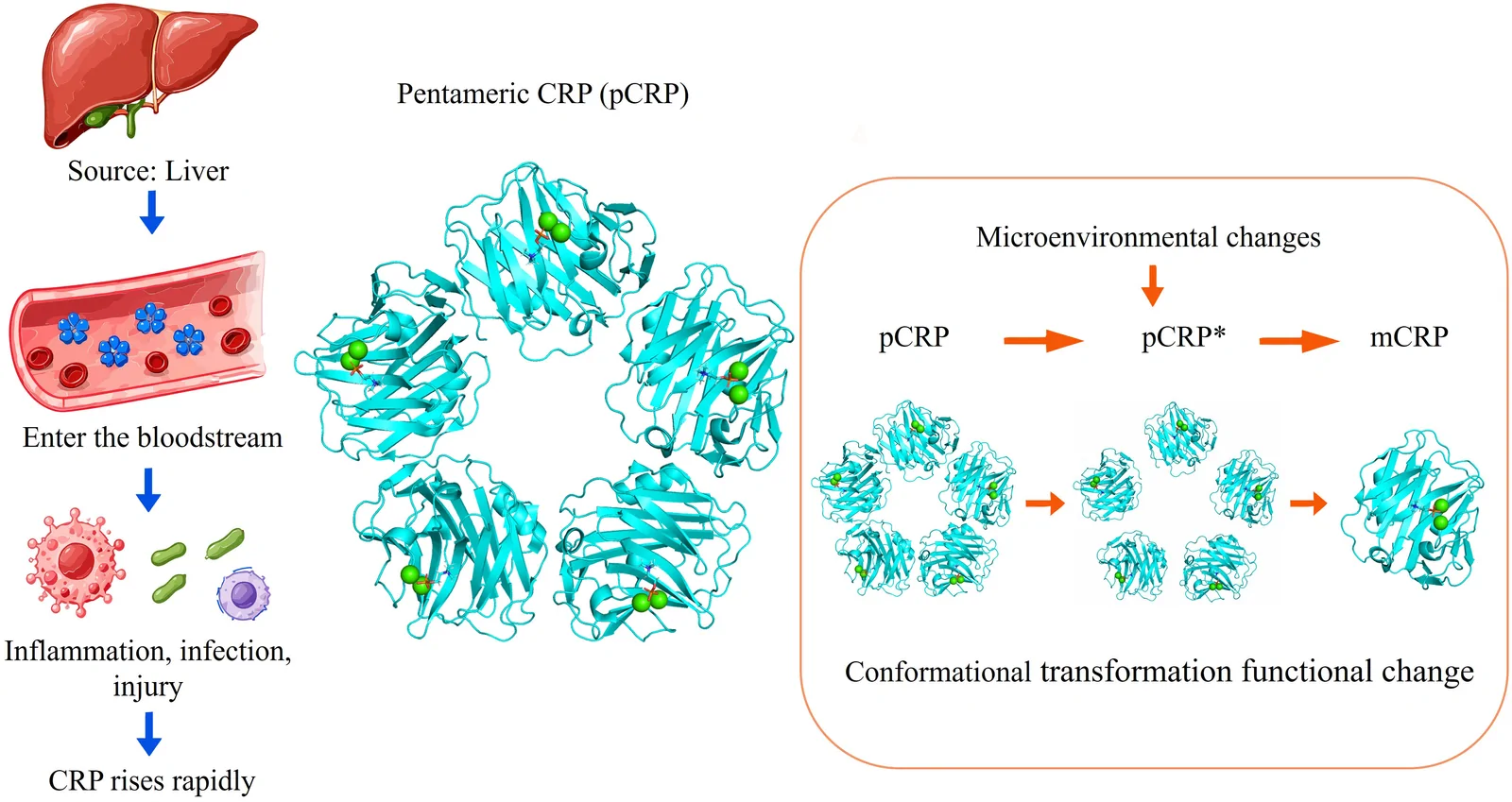
The production, circulation, and conformational changes of CRP are subject to influence from its microenvironment. Activated cell membranes, damaged membrane surfaces, or a local inflammatory microenvironment induce interactions between pCRP subunits to gradually weaken, ultimately leading to the formation of an activated or partially dissociated pentameric intermediate state, termed pCRP*. In this state, pCRP retains its pentameric conformation, yet with expanded distances between subunits, looser packing, and exposure of binding regions and novel epitopes typically hidden in native pCRP. Disruption of noncovalent interactions between subunits results in complete dissociation of pCRP into mCRP, which lacks the pentameric ring structure, exposing more hydrophobic regions and conformational epitopes. As a result, mCRP typically shows increased membrane-binding capacity and pro-inflammatory activity. The gradual transition of pCRP from pCRP* to mCRP is depicted by the orange solid arrow in the image, with the green spheres representing calcium ions.

## 2 Structural and functional characteristics of CRP

### 2.1 Structure basis of CRP

The molecular weight of each CRP subunit is approximately 23 kDa, consisting of 206 amino acid residues, resulting in a total molecular weight of around 115 kDa for pCRP (10, 14) (**Figure 2**). pCRP oligomerizes into a ring-shaped pentamer via hydrophobic interactions, hydrogen bonds, and electrostatic interactions at subunit interfaces, forming a disc-like molecule with a central cavity (15–17). The stability of the pentamer, its capacity for conformational changes, and its ability to convert to monomeric CRP (mCRP) in inflammatory conditions primarily stem from noncovalent interactions (18). This conformation arranges the five B-face ligand binding sites in an orderly manner on one side, facilitating CRP’s attachment to surfaces with multivalent ligands. Located on the opposite side, the A-face acts as a binding platform for effector molecules such as C1q and Fcγ receptors, spatially segregating the roles of recognition and effector functions.

**Figure 2:**
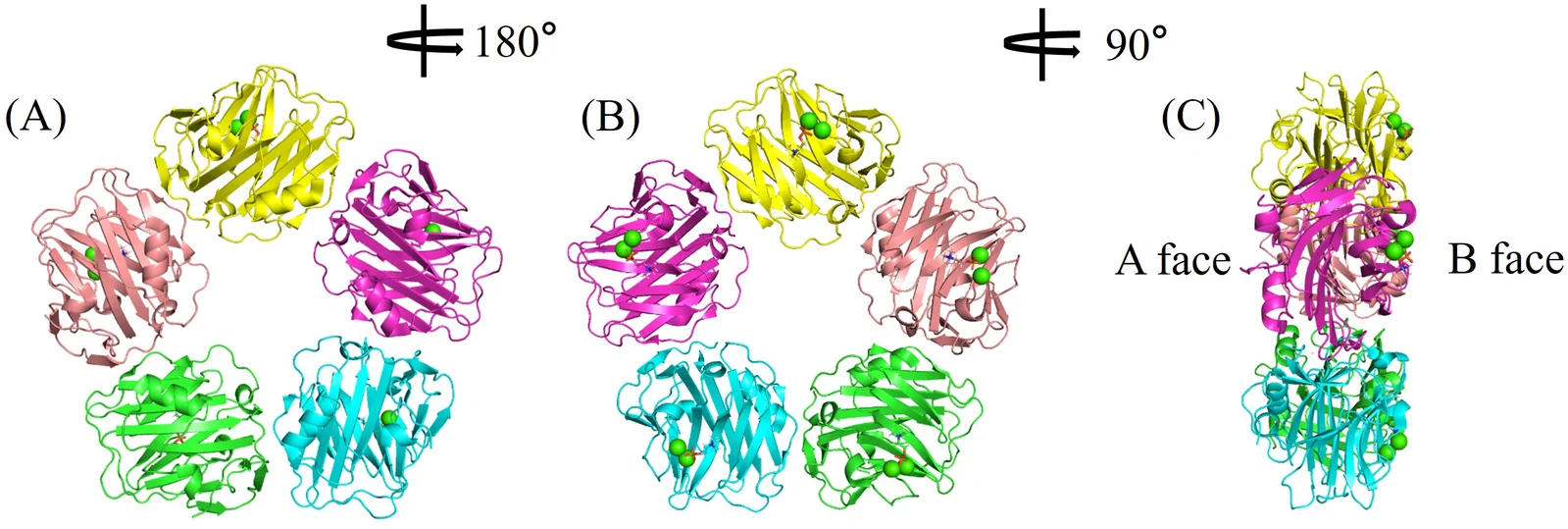
Schematic representation of CRP structure from various perspective. **(A)** Effector face (A-face) diagram; **(B)** Recognition face (B-face) diagram, derived from image **(A)** by a 180° vertical rotation; **(C)** Side view diagram, obtained by a 90° horizontal and 180° vertical rotation of image **(A)**. The depiction includes green spheres symbolizing calcium ions.

Essential to the structure and function of CRP are its two conserved short motifs: the PC-binding site (10, 19) and the C1q-binding site (12, 20). The PC binding site includes critical residues such as Phe66, Trp67, and Glu81, where Phe66 plays a direct role in ligand recognition, Glu81 is closely associated with Ca^2+^ binding, and Trp67 primarily maintains the spatial conformation stability of the binding pocket. These residues collectively establish a calcium-dependent ligand-binding environment (2, 7). The C1q-binding site situated on the A-face of each CRP subunit comprises residues such as Asp112, Glu88, and Tyr175, which are involved in C1q recognition. This site mediates the activation of the classical complement pathway by binding to the globular head region of C1q (12, 21).

The CRP subunit does not adopt a linear peptide chain conformation but forms a compact spherical structure with a well-defined spatial orientation. Its core comprises a typical β-sandwich fold, consisting of two layers of antiparallel β-sheets that establish a stable hydrophobic core framework (13, 22). The conserved disulfide bond between Cys36 and Cys97 is critical for the correct folding and local stability (23, 24). The ligand binding pocket on the B-face is shaped by Ca^2+^-coordinating and hydrophobic residues, along with adjacent ring regions. Key residues such as Glu81 and Phe66 are in close spatial proximity, facilitating phosphate group anchoring, choline group recognition, and local charge stabilization within the same three-dimensional environment (24, 25).

### 2.2 Functional differentiation of CRP surfaces

The two planes of the pCRP exhibit significant structural and functional differences, essential for its role as a bridging molecule between the components of the innate immune system (**Figure 3**). The B-face serves as the main functional interface for CRP to attach to ligands. This region contains two Ca^2+^-binding sites within each subunit and forms, together with adjacent hydrophobic pockets, a specialized environment for ligand binding, facilitating high-affinity interaction with molecules like PC (26, 27). As all five subunits’ ligand-binding sites are situated on the B-face, CRP can bind multiple ligand molecules simultaneously, thereby enhancing its binding capacity and opsonization efficiency on polyvalent surfaces, such as bacterial capsules and apoptotic cell membranes (28, 29).

**Figure 3:**
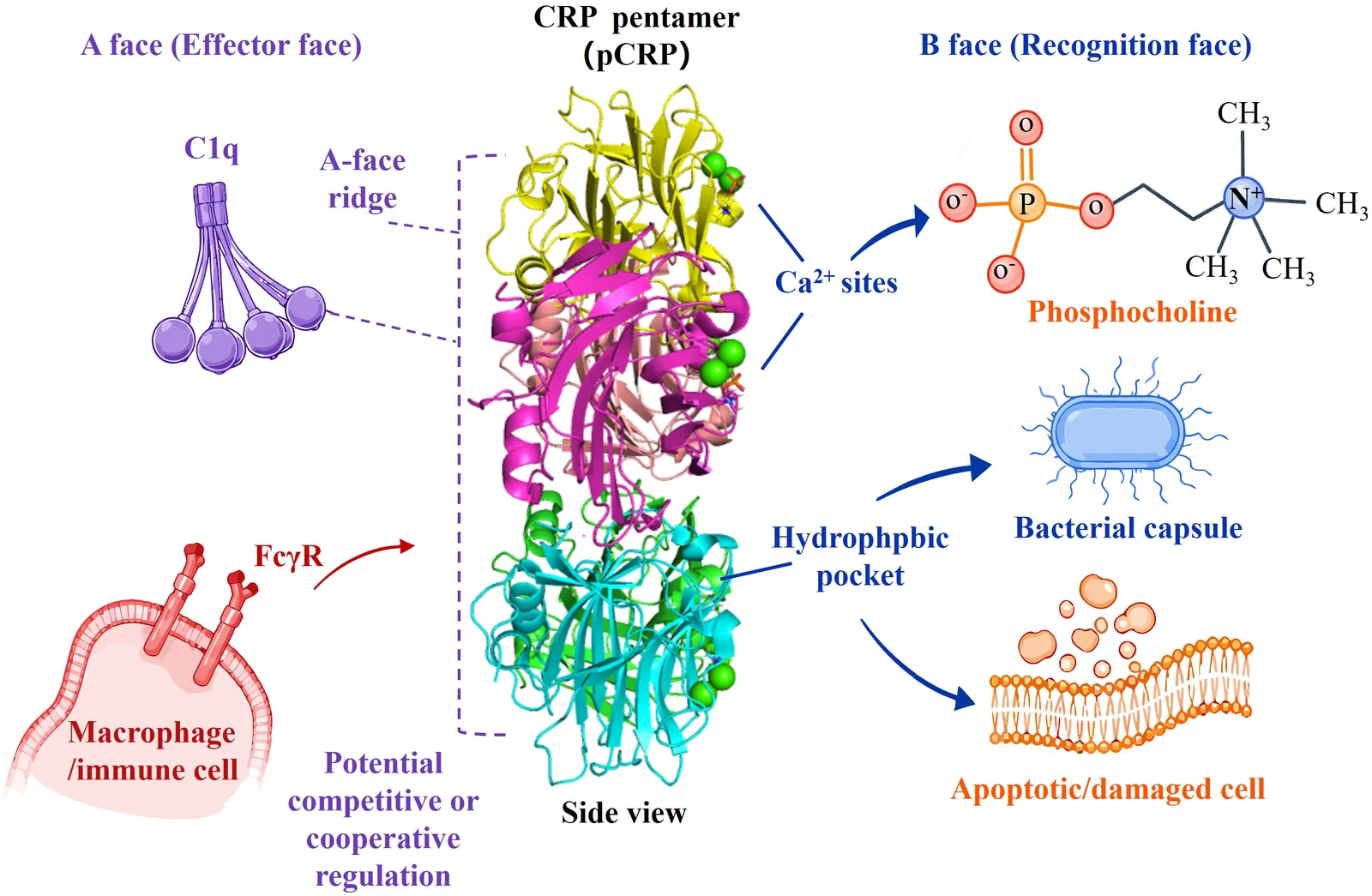
Schematic diagram showing the structural compartments and functional differentiation of the A-face and B-face of the CRP pentamer. The A-face, located on the left, function as the effector face. Its A-face ridge can bind to complement C1q, facilitating activation of the classical complement pathway, and may interact with FcγR on immune cell surfaces, influencing phagocytosis and inflammation regulation. The purple dashed line represents the C1q-A-face interaction, while the red arrow indicates the functional connection between the A-face and FcγR. The B-face situated on the right, serve as the recognition face, housing two Ca^2+^-binding sites and an adjacent hydrophobic pocket. This configuration allows for Ca^2+^-dependent recognition of phosphatidylcholine, bacterial capsules, and apoptotic or damaged cell membranes. The green spheres represent Ca^2+^, and the blue arrows signify the recognition interaction between the B-face and different ligands.

The A-face predominantly facilitates the interaction between CRP and downstream effector molecules. It features a multi-residue cooperative interaction interface at its core, primarily formed by the A-face ridge region rather than a single secondary structural element. This region, through its spatial conformation, serves as the main binding interface with the spherical head of complement C1q, crucial for activation of the classical complement pathway (30, 31). Moreover, the A-face participates in interactions with FcγRI and other Fc receptor-associated pathways on immune cell surfaces, thereby regulating opsonized phagocytosis and inflammatory amplification. Recent research indicates potential competitive or synergistic regulatory mechanisms at the functional level due to partial spatial overlap or conformational coupling between C1q and FcγR at the CRP-binding interface (32, 33).

The spatial segregation of the A-face and B-face enables CRP to achieve a high degree of structural-functional coupling: After recognizing ligands on pathogen or damaged cell surfaces through the B-face, membrane-associated CRP can undergo conformational rearrangement that alters the accessibility of its effector face and thereby facilitates interactions with complement components such as C1q. This process enhances the recognition and removal of targets by phagocytes, enabling precise regulation of the immune response to external stimuli or injuries. Such spatial compartmentalization minimizes the risk of unintended activation of self-tissues (34, 35).

### 2.3 Complement activation by CRP

During infection, tissue damage, or inflammation, CRP binds specifically to PC on apoptotic/necrotic cells, bacterial cell walls (e.g., *Streptococcus pneumoniae*) (25, 36) and the interactions with modified low-density lipoprotein (LDL) have also been reported, although their magnitude and specificity appear to depend strongly on the conformational state of CRP and the physicochemical characteristics of the LDL preparation. This binding is essential for CRP to initiate subsequent immune responses. Upon binding to a PC-containing ligand, CRP undergoes a local conformational change, tilting or altering its pentameric structure to expose a previously hidden active surface. This surface contains binding sites for complement component C1q, which activates C1r and C1s to initiate the classical complement pathway (12). Studies have identified Asp112 and Tyr175 residues on CRP as crucial for direct binding to C1q, enabling the transformation of CRP from an inert circulating protein to an activated molecule capable of initiating the complement cascade (30).

Activated CRP binds to C1q via its exposed binding surface. Previous studies suggest that CRP recognition predominantly involves the positively charged residue region in the globular head of the C1q A chain, while other ligands like IgG1 and PTX3 rely more on other C1q chains or varied surface residues (37). Thus, the spherical head of C1q can be regarded as a multi-ligand recognition platform that achieves discriminatory binding to CRP, immunoglobulins, and other pattern recognition molecules through diverse residue combinations (38, 39).

The interaction between CRP and C1q exhibits distinct charge dependence and regional selectivity. It primarily involves electrostatic attraction between the negatively charged regions on the A-face of CRP and the positively charged residue regions within the globular head module of the C1q A chain (39). Although both CRP-C1q and IgG-C1q recognition are strongly influenced by electrostatic interactions, their interfacial architectures appear to differ markedly. On the CRP A-face, Asp112 represents a key negatively charged determinant within the extended effector-face cleft, whereas Tyr175 contributes to hydrogen-bonding interactions and Lys114 may modulate accessibility to the C1q-binding region. Structural models position the predominantly basic crown of the globular head of C1q toward the negatively charged central region of the pCRP, with CRP Tyr175 lying within hydrogen-bonding distance of Tyr175 in the C1q B chain and Lys200 in the A chain. Mutational studies further implicate C1q residues such as TyrB175 and LysC170 in CRP recognition, indicating that the interface is stabilized by a combination of electrostatic complementarity, hydrogen bonding, and hydrophobic contacts rather than by a single dominant salt bridge (30, 39, 40).

Although quantitative measurements of the energetic contribution of individual CRP-C1q contacts remain limited, available structural and mutational evidence suggests that CRP recognition is mediated by distributed charge complementarity across the A-face cleft rather than by a single high-affinity ionic interaction (12, 41, 42). By contrast, classical IgG-C1q recognition is centered largely on the B-chain globular head module of C1q (ghB), particularly its positively charged Arg-rich surface. In proposed structural models, IgG Asp270 and Lys322 form complementary salt bridges with C1q ArgB129 and GluB162, respectively, while ArgB114, ArgB161, and HisB117 provide additional stabilizing contacts. Thus, IgG-C1q binding appears to rely on a comparatively well-defined ionic network between the Fc CH2 domain and ghB, whereas CRP-C1q recognition involves a broader and more asymmetric electrostatic interface extending across the CRP effector-face cleft and multiple C1q globular-head modules. These differences may contribute to the distinct geometrical requirements and complement-activation properties of CRP- and immunoglobulin-mediated C1q engagement (43).

While the binding of CRP to C1q relies on Ca^2+^ for maintaining its interaction with the ligand, C1q self-assembly is not Ca^2+^-dependent (44). Effective complement activation may involve the aggregating multiple pCRP on the ligand surface to provide a stable platform for C1q binding (31). The classical complement pathway activated by CRP tends to promote controlled activation, favoring the formation of C3 convertase and C3b deposition over efficient progression to C5 convertase and the membrane attack complex. This process aids in target clearance while limiting excessive inflammatory damage (45).

## 3 Calcium-dependent structural maintenance and ligand recognition of CRP

### 3.1 Calcium-binding pocket

Calcium ions play a crucial role in both the structural and functional regulation of CRP. They not only provide stability but also directly participate in the formation and regulation of ligand binding sites. Each CRP subunit precisely binds two Ca^2+^ ions in the ligand binding region, collectively maintaining the geometric configuration of the binding pocket on the B-face and improving ligand recognition, such as PC (13, 28). One of the Ca^2+^ sites (Ca1) is primarily coordinated by multiple acidic amino acid residues and main-chain carbonyl oxygen atoms, resulting in an octahedral geometry. This coordination environment stabilizes the ligand binding conformation and facilitates ligand-protein interactions. The coordination of Ca^2+^ involves conserved residues, such as Asp60, Asn61, Glu138, and Asp140, along with the main-chain carbonyl oxygen of Phe66. The remaining coordination sites are occupied by structural water molecules, thereby forming a stable coordination network characterized by octahedral geometry (13, 46, 47).

The second Ca^2+^ (Ca2) binding site exhibits a coordination pattern akin to Ca1, involving residues Gln139, Glu147, and Gln150. Asp140 contributes bidentate coordination, enhancing the local structure’s stability. The conformational integrity of the ligand binding pocket and its Ca^2+^-dependent recognition ability are thus maintained synergistically. Glu81 is pivotal in the calcium-binding network of CRP, aiding in local coordination and forming a structural link between Ca1 and Ca2, thereby partially stabilizing the dual-calcium-binding system (46, 48) (**Figure 4**; **Table 1**).

**Figure 4:**
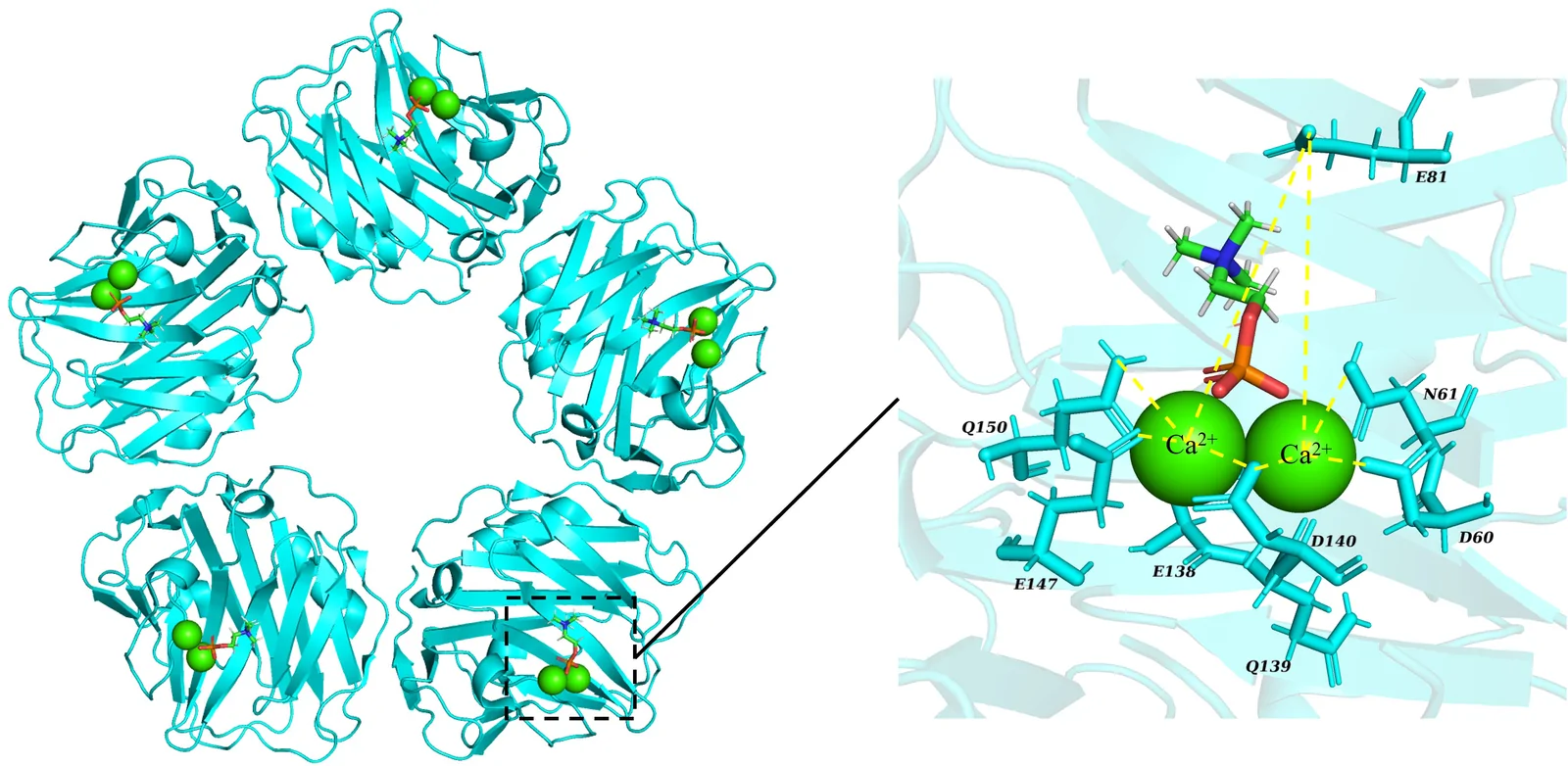
Calcium ion-binding pockets. The left panel shows a top-down view of pCRP, exhibiting five identical subunits arranged in a circular formation. Green spheres symbolize Ca^2+^-binding sites spread across the B-face of each subunit, with a magnified region highlighted by a black box. The right panel displays an expanded perspective of the Ca^2+^-dependent ligand binding pocket within a single subunit, featuring two Ca^2+^ ions (Ca1 and Ca2) situated in the phosphocholine-binding area. These ions engage in a coordination network with specific residues such as Asp60, Asn61, Glu138, Asp140, and nearby main-chain atoms. Residues including Glu81, Gln139, Glu147, and Gln150 surround the binding pocket, collectively contributing to local conformational stability and ligand recognition. Yellow dashed lines denote interactions between Ca^2+^ coordination or ligands and the binding sites. This dual-Ca^2+^ network plays a crucial role in preserving the integrity of the binding pocket on the B-face and enhancing the Ca^2+^-dependent recognition of phosphocholine ligands by CRP.

**Table 1:** Amino acids involved in calcium ion binding.

Calcium ions	Coordinating residues	Functional characteristics of Ca^2+^
First calcium ion (Ca1)	Asp60, Asn61, Glu138, Asp140, Glu81	Situated deep within the pocket, it is tightly locked in place by acidic/polar residues and the carbonyl oxygen of the main chain
Second calcium ion (Ca2)	Gln139, Glu147, Gln150, Asp140, Glu81	Situated on the external aspect of the pocket, Gln150 is pivotal in the subsequent recognition of ligands
Common residues	Asp140, Glu81	The central bridge, binding Ca1 and Ca2 with its side chains simultaneously

The Ca^2+^ site should be viewed as a dynamic coordination environment rather than a rigid structural motif. Upon initial ligand encounter, the phosphate moiety of phosphocholine is likely to engage the Ca^2+^-containing recognition site first, allowing local rearrangement of the Ca^2+^-coordinating network formed by residues including Asp60, Asn61, Glu138, Gln139, Asp140, Glu147, and Gln150 (25, 49). Such coordination adjustments may facilitate optimal positioning of the ligand phosphate group between the two Ca^2+^ ions. Following this initial Ca^2+^-mediated recognition, the phosphocholine headgroup becomes more fully anchored as its choline moiety is accommodated within the adjacent hydrophobic pocket centered on Phe66 and further stabilized by interaction with Glu81 (22). This Ca^2+^-dependent ligand recognition process provides the structural basis for subsequent interactions of CRP with phosphocholine-containing biological surfaces, including lysophosphatidylcholine-enriched membranes, where membrane engagement can promote further conformational remodeling toward activated CRP states (50, 51). Thus, Ca^2+^ may function not only as a static structural cofactor but also as a dynamic coordination bridge that couples initial phosphate recognition to the subsequent stabilization of phosphocholine within the ligand-binding pocket. However, the precise sequence and kinetics of these early coordination rearrangements remain to be resolved experimentally.

### 3.2 Structural stabilization effect mediated by Ca²^+^

Calcium ions are crucial for maintaining the structural stability of the CRP pentamer. They stabilize the conformation of the local ring region by neutralizing the negative charges of acidic residues near the binding pocket on the B-face. This action preserves the integrity and rigidity of the ligand binding region (52, 53). In the absence of Ca^2+^, the conformational stability of these ring regions decreases significantly, leading to increased local flexibility and conformational relaxation. This weakening effect compromises the overall structural integrity of the B-face, potentially initiating the dissociation process of the pentamer (46). Therefore, the loosening of the B-face binding region can impact the subunit-subunit interaction network through conformational coupling, diminishing the overall thermodynamic stability of the pentamer. This makes the pentamer more vulnerable to conformational rearrangement or dissociation in specific microenvironments like those with low calcium levels, acidity, or membrane surfaces (54).

In low-calcium environments or in the presence of metal ion chelators such as ethylenediaminetetraacetic acid (EDTA), Ca^2+^ gradually dissociates from the CRP binding site, leading to increased flexibility in the ring region of the ligand binding pocket on the B-face and local structural relaxation (55). This process significantly reduces or nearly eliminates CRP’s ability to recognize classic ligands such as PC (13, 22, 54). This conformational change impairs CRP’s pattern recognition function and may disrupt the noncovalent network of inter-subunit interactions, including hydrophobic interactions, hydrogen bonds, and electrostatic interactions, further reducing the stability of the pentamer (24, 38, 56). The decreased stability of the pentamer gradually disrupts its strict C5 symmetry, setting the stage for subsequent CRP conformational transitions induced by the inflammatory microenvironment.

Molecular dynamics studies have demonstrated the utility of computational approaches for resolving the energetic basis of macromolecular stability and intermolecular interactions. Parameters including root-mean-square deviation and fluctuation, radius of gyration, principal component analysis, free-energy landscapes, and residue-interaction networks can collectively reveal how changes in non-covalent contacts, ionic interactions, and conformational flexibility influence protein stability (57). In this framework, Ca^2+^ depletion in CRP may be interpreted as a perturbation of the interaction network that stabilizes the pentamer, potentially increasing conformational mobility, weakening inter-subunit contacts, and reshaping the free-energy landscape toward partially destabilized or dissociation-prone states. Although direct molecular dynamics simulations of the complete Ca^2+^-depletion-induced pCRP-to-mCRP transition remain limited, integrating atomistic simulations with structural and biochemical data could provide mechanistic insight into the energetic pathways and intermediate states associated with pentamer destabilization (13, 56).

### 3.3 Calcium-dependent ligand-binding of CRP

Calcium ions play a crucial role in facilitating the interaction between CRP and its ligand. The negative charge of phosphate groups on the PC molecule hinders direct stable interactions with the acidic residues in the binding pocket. However, Ca^2+^ counteracts this electrostatic repulsion by simultaneously coordinating with the phosphate oxygen atoms of PC and residues such as Glu81 within the binding pocket. Thus, Ca^2+^ acts as a cationic bridge, enhancing PC recognition and anchoring the ligand within the Ca^2+^-dependent binding pocket on the B-face (22, 46). Additionally, Ca^2+^ contributes to maintaining the correct spatial configuration of the binding pocket, allowing hydrophobic residues such as Phe66 to engage in hydrophobic and cation-π interactions with the choline moiety of PC. This interaction mechanism enables CRP to recognize PC with high affinity and molecular-level specificity (58).

The PC-binding site is situated in a shallow pocket on the B-face of each CRP subunit. The phosphate group of PC is deeply positioned within this pocket, forming a stable coordination network with conserved residues. The choline moiety, on the other hand, is nestled in a hydrophobic environment created by aromatic and aliphatic residues. Key residues such as Phe66, Leu64, and Glu81 play essential roles in shaping and stabilizing the ligand-binding pocket (10, 25, 59).

CRP, in addition to PC, has the ability to identify various endogenous ligands with PC-like molecular patterns, such as oxidized phospholipids on apoptotic and necrotic cell membranes, modified lipoproteins, and specific nuclear-associated antigens. The interaction between CRP and modified LDL, particularly oxidized LDL (oxLDL), warrants careful interpretation, as LDL-binding behavior may vary among different CRP conformations and can also be influenced by protein purification, storage, and handling conditions. Early studies reported that CRP binds oxLDL through phosphorylcholine-containing epitopes exposed during phospholipid oxidation (8, 59, 60). However, subsequent side-by-side analyses of distinct CRP conformations indicated that native pentameric CRP exhibits little or markedly weaker binding to native or oxidized LDL, whereas structurally modified CRP displays substantially enhanced LDL-binding activity. Importantly, even a minor proportion of conformationally altered CRP within an apparently pentameric preparation may contribute detectable LDL-binding activity. These findings suggest that discrepancies among earlier studies may partly reflect differences in CRP preparation, purification, storage conditions, and conformational homogeneity, rather than an intrinsic strong interaction between native pCRP and oxLDL. Therefore, reported CRP–oxLDL interactions should be interpreted in the context of both the conformational state of CRP and the physicochemical characteristics of the modified LDL preparation (8, 61).

At the molecular level, CRP recognition of oxLDL is mediated predominantly by oxidized phosphatidylcholine species in which oxidative remodeling exposes the phosphocholine headgroup for engagement with the Ca^2+^-dependent binding pocket on the B-face. Competition studies indicate that these oxidized phosphatidylcholine species can compete effectively with phosphocholine-containing ligands for CRP binding, whereas non-oxidized phosphatidylcholine and oxidized phospholipids lacking a phosphocholine headgroup exhibit little or no competitive activity. Thus, oxidation-associated phosphocholine epitopes represent major CRP-reactive determinants within oxLDL (8, 62). Importantly, the principal recognition determinant is not the oxidized acyl chain itself, but the phosphocholine moiety that becomes sterically accessible following lipid oxidation. During atherogenesis, the accumulation of these exposed phosphocholine epitopes on LDL may provide multivalent ligands for the same Ca^2+^-dependent B-face pocket involved in conventional phosphocholine recognition, thereby promoting CRP recruitment to oxidatively modified lipoproteins (63).

## 4 Factors influencing conformational transitions and functional differentiation in CRP

### 4.1 Ca²^+^ depletion-driven dissociation

Disruption of Ca^2+^ homeostasis, in addition to external microenvironmental stimuli, is a crucial factor that regulates the conformational dynamics of CRP (34). CRP typically exists in a stable pentameric form (pCRP), under normal conditions, with the Ca^2+^-bound state being vital for maintaining its conformational stability. The stability of the CRP pentamer diminishes gradually when local Ca^2+^ availability decreases or Ca^2+^ binding is disrupted, prompting a transition towards more flexible conformational states (64).

EDTA is commonly employed to chelate Ca^2+^ and mimic Ca^2+^-depleted conditions to investigate CRP conformational changes (65). By reducing the availability of Ca^2+^, EDTA disrupts Ca^2+^-dependent structural stabilization, leading to local conformational relaxation of CRP. This results in decreased ligand-binding capacity and pentamer stability. The structural perturbations induced by Ca^2+^ loss may propagate to inter-subunit interfaces. Disruption of Ca^2+^-mediated stabilization reduces inter-subunit cooperativity and increases conformational flexibility. Rather than immediate dissociation, this process may represent a continuous conformational transition from stable pCRP toward the activated intermediate pCRP*. This transition involves rearrangement of subunit orientations, exposure of previously buried epitopes and hydrophobic regions, providing a structural basis for subsequent mCRP formation (38).

With the gradual weakening of inter-subunit noncovalent interactions, pCRP undergoes further dissociation into mCRP. This transition exposes previously hidden structural regions, enhancing membrane-binding capacity and pro-inflammatory activity. It signifies a shift in CRP functionality from a systemic homeostatic form to a locally acting inflammatory mediator. The loss of Ca^2+^ does not directly disassemble the CRP pentamer but disrupts the Ca^2+^-mediated conformational stabilization network, compromising pentamer integrity. This loss increases CRP’s vulnerability to various environmental factors such as acidic conditions, oxidative stress, and membrane lipid signals. Consequently, this facilitates the conversion of pCRP to the activated intermediate pCRP* and eventually to mCRP (34).

A central question in CRP conformational biology is whether pCRP can undergo structural conversion under the Ca^2+^-rich conditions of the extracellular environment, rather than simply whether dissociation can occur after Ca^2+^ depletion. Early studies showed that removal of Ca^2+^ markedly destabilizes CRP and promotes the formation of structurally and functionally distinct monomeric species (66). In contrast, Ca^2+^-bound pCRP is highly resistant to conformational perturbation, which long raised doubts about the physiological relevance of CRP dissociation observed under Ca^2+^-depleted conditions. This apparent contradiction contributed to persistent uncertainty over whether mCRP could be generated from circulating pCRP *in vivo*. The key unresolved issue therefore became whether a physiologically relevant mechanism could drive CRP conformational remodeling in the continued presence of Ca^2+^, without relying on artificial Ca^2+^ depletion or chemical denaturation (67).

The concept of CRP conformational conversion developed from early observations that removal of Ca^2+^ destabilizes native pCRP and promotes the formation of structurally altered or monomeric CRP species. Subsequent studies demonstrated that these non-native CRP conformations possess biological activities distinct from those of native pCRP, raising the possibility that structural conversion may contribute to CRP functional diversification (60, 68). However, Ca^2+^-bound pCRP is highly conformationally stable, whereas extracellular CRP normally exists in a Ca^2+^-rich environment. This apparent contradiction led to longstanding uncertainty regarding whether pCRP-to-mCRP conversion could occur under physiologically relevant conditions, a concern reflected in critical assessments of CRP biology in the early 2000s. A major conceptual advance came with the demonstration that Ca^2+^-dependent binding of native pCRP to phosphocholine-containing membranes could itself induce partial structural remodeling while the pentameric architecture was initially retained, followed by further conformational conversion. This provided a physiologically plausible route by which circulating pCRP could undergo localized structural activation in the continued presence of Ca^2+^ and established membrane-associated conformational remodeling as a mechanistic framework for subsequent studies of CRP structural transitions (50, 69).

### 4.2 Microenvironment-driven conformational transition and functional differentiation

Research indicates that CRP conformational remodeling can occur in the continued presence of Ca^2+^. Ca^2+^-dependent recognition of membrane-associated phosphocholine-containing ligands provides a physiological route for recruiting native pCRP to damaged or activated membranes, where structural conversion can subsequently occur. Early studies provided direct evidence that pCRP binds model membranes and apoptotic cell surfaces under Ca^2+^-containing conditions and undergoes progressive remodeling, with lysophosphatidylcholine (LPC) acting as a key membrane determinant. These findings provided a plausible mechanism for local mCRP generation *in vivo* and established membrane binding as a physiologically relevant pathway for CRP conformational activation rather than merely another destabilizing stimulus. Once a membrane-associated pathway for Ca^2+^-permissive CRP conversion had been established, subsequent studies further demonstrated that local physicochemical conditions can modulate the stability and progression of this conformational pathway (70).

Environmental factors collectively facilitate the transition of pCRP from a stable circulating state to an activated conformational state. Local pH changes act as important signals driving CRP conformational transitions (71, 72). Inflamed or ischemic tissues are often characterized by lactic acid accumulation and enhanced proton release, resulting in the establishment of a locally acidic microenvironment with pH values that may decrease to approximately 5.5-6.5 (73).

At mildly acidic pH, protonation of ionizable residues is likely to contribute to the destabilization of the pCRP pentamer by altering local electrostatic interactions and hydrogen-bonding networks at and near the subunit interfaces. Histidine residues are of particular interest because their side-chain pK_a_ values lie close to the pH range encountered in inflammatory microenvironments, allowing their protonation states to respond sensitively to relatively modest decreases in pH. Structural studies have identified His95 as a potential pH-sensitive site, with low-pH conditions associated with rearrangement of the local hydrogen-bonding network involving His95, a conserved water molecule, and residues adjacent to the Cys36–Cys97 disulfide bond (31). Such changes may increase local conformational flexibility and facilitate subsequent structural remodeling. However, current evidence does not establish histidine protonation as the sole or primary trigger of pCRP destabilization. Protonation of acidic residues, particularly Asp and Glu side chains involved in inter-subunit electrostatic contacts, may also weaken salt bridges and hydrogen bonds that stabilize the pentamer. These pH-induced local perturbations may reduce the energetic stability of the pentameric architecture and lower the barrier for subsequent conformational remodeling. Although acidic pH alone is unlikely to be sufficient to drive complete pCRP-to-mCRP conversion, it may facilitate the formation of intermediate conformational states by increasing structural flexibility and exposing regions that become responsive to additional environmental triggers, such as membrane interaction. This concept is consistent with the broader model that CRP conformational transitions are regulated by a combination of local physicochemical cues rather than by a single molecular switch (51, 70). Thus, acidic pH is more appropriately viewed as inducing a coordinated redistribution of protonation states and electrostatic interactions, with histidine protonation potentially acting as one component of a broader pH-sensitive destabilization mechanism rather than as an isolated molecular switch (71).

High-resolution cryo-EM and biochemical studies have revealed that a decrease in pH to acidic levels leads to structural rearrangements in surface charge distribution, local hydrogen-bond networks, and relative subunit orientations (31). These modifications impact the stability of the pentameric assembly and its ligand-binding properties (74). Protonation causes modifications in the electrostatic properties and hydrogen-bond networks of key residues, thereby compromising the stability of protein loop regions and inter-subunit interfaces where these residues are located. These local perturbations induce subtle rearrangements of the overall pentameric architecture and weaken inter-subunit interactions (75).

The pH-induced conformational transition signifies the creation of a functionally preactivated CRP state, rather than a simple dissociation process (76). Under acidic conditions, CRP shows increased affinity for C1q, suggesting a heightened potential for classical complement activation at inflammatory sites (39). CRP binding to IgG and IgM is also facilitated in acidic environments, potentially augmenting its opsonic clearance function. Furthermore, acidic pH levels can impact the assembly of CRP into higher-order structures, such as the formation of decamers composed of two pentamers arranged face-to-face, under specific experimental conditions (31).

Microparticles released from damaged cell membranes or activated cells exhibit an enrichment of LPC, which structurally resembles PC and can bind to the Ca^2+^-dependent ligand-binding pocket on the B-face of CRP (77). Upon binding to an LPC-rich membrane surface, circulating pCRP undergoes a localized conformational rearrangement, leading to the formation of an activated intermediate conformation termed membrane-associated intermediate. While maintaining its pentameric assembly, membrane-associated intermediate experiences weakened inter-subunit interactions, a more relaxed overall conformation, and partial exposure of previously hidden epitopes and hydrophobic regions, thereby enhancing membrane interactions. Consequently, membrane-associated intermediate is identified as a transitional intermediate state in the conversion process from pCRP to mCRP.

*In vitro* studies have shown that LPC-rich micelles facilitate the conversion of pCRP to mCRP and promote the delivery and deposition of mCRP onto vascular endothelial cell surfaces, leading to endothelial cell activation (**Figure 5**). Additionally, research indicates that patients with myocardial infarction have elevated levels of mCRP on circulating micelle surfaces, while healthy individuals exhibit significantly lower mCRP levels (78). These findings suggest that the microenvironment associated with membranes plays a crucial role in triggering CRP conformational conversion (77). According to this conformational transition process, pCRP, membrane-associated intermediate, and mCRP represent distinct functional states within the CRP conformational spectrum, each possessing unique structural and biological characteristics (79).

**Figure 5:**
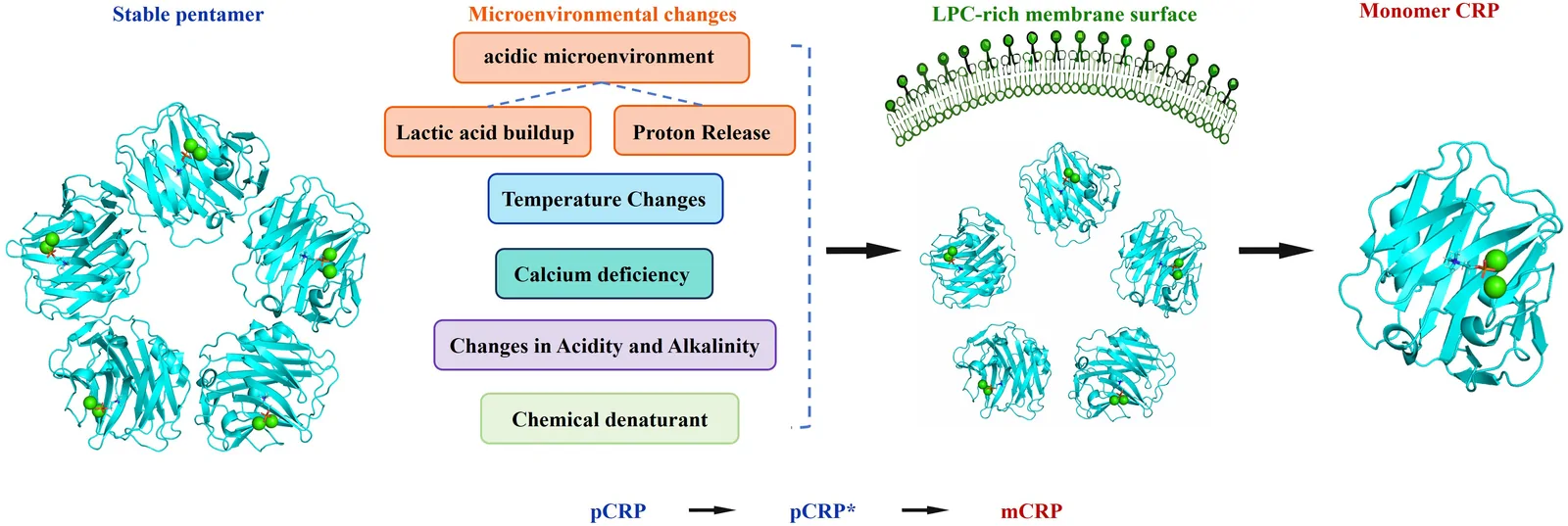
Environmental factors impact CRP allosterism and activity. Lactic acid accumulation, proton release, pH variations, and temperature variations in inflamed or damaged tissues can disturb surface charge, hydrogen-bond networks, and inter-subunit interactions of pCRP. Interaction with LPC-rich activated or damaged cell membranes induces localized conformational changes in pCRP, leading to the formation of the activated pentameric intermediate pCRP*. While retaining its pentameric structure, pCRP* undergoes relaxation in subunit interfaces, revealing concealed epitopes. Subsequent weakening of inter-subunit interactions results in the dissociation of pCRP* into mCRP. The progression of CRP from stable pCRP, through pCRP*, to mCRP is illustrated by the depicted arrows in the image. The green spheres in the diagram represent calcium ions.

When pCRP binds to activated platelets, damaged cell membranes, or LPC-rich membrane surfaces, a local conformational rearrangement may first occur, forming the activated pentameric intermediate pCRP* (56, 80). Compared with native pCRP, pCRP* retains the overall pentameric framework but exhibits weakened inter-subunit interactions and increased subunit separation. Local changes in pentameric symmetry and subunit interfaces result in partial exposure of previously buried epitopes and hydrophobic regions. This conformation exhibits enhanced membrane-binding affinity and local retention capacity, with its biological phenotype shifting from a predominantly regulatory circulating state toward a more pro-inflammatory state. Furthermore, pCRP* provides the structural basis for subsequent pentamer dissociation and mCRP formation (81).

As noncovalent interactions between subunits become further disrupted, pCRP can dissociate into mCRP. Monomer CRP is predominantly detected in localized inflammatory microenvironments, including vascular endothelium, activated platelets, atherosclerotic plaques, and damaged cell membranes (82, 83). Compared with pCRP, mCRP loses its pentameric symmetry and exposes previously concealed epitopes and hydrophobic regions that are buried at the subunit interfaces, including the cholesterol-binding sequence near the N-terminus and other cryptic ligand-binding regions (84, 85). These structural rearrangements enable mCRP to interact with diverse ligands in a Ca^2+^-independent manner (86, 87). mCRP exhibits enhanced capacity to activate endothelial cells and promotes the expression of adhesion molecules (27, 88). Furthermore, mCRP can act directly or indirectly on leukocytes, facilitating the adhesion, chemotaxis, migration, and activation of neutrophils and monocytes, thereby enhancing leukocyte recruitment and reactive oxygen species production at inflammatory sites. Thus, the transition from pCRP to mCRP represents a functional shift from a systemic immune surveillance molecule to a local inflammatory amplifier (35, 51, 89).

The kinetics of CRP conformational conversion are highly dependent on the local physicochemical environment and therefore do not follow a uniform timescale. Under physiological conditions, circulating pCRP is relatively stable and undergoes limited spontaneous dissociation, consistent with the predominance of the native pentameric isoform in plasma (34, 44). By contrast, pathological or experimentally destabilizing conditions can markedly accelerate conformational remodeling. Under strong experimental perturbation, such as chemical denaturation combined with Ca^2+^ depletion, pCRP can rapidly dissociate into monomeric subunits within minutes. Lipid microparticles have likewise been shown to promote pCRP-to-mCRP dissociation within approximately 20 min, indicating that membrane-associated environments can efficiently facilitate conformational conversion. Under conditions that more closely resemble inflammatory microenvironments, however, the process generally occurs over several hours. For example, combined oxidative stress and mildly acidic pH can induce substantial CRP dissociation within approximately 4 h, whereas considerably less dissociation occurs under physiological pH conditions (71).

Within this broader kinetic framework, membrane-associated conversion is of particular mechanistic significance because it demonstrates that CRP remodeling can proceed in the continued presence of Ca²^+^. Rather than representing a simple two-state dissociation, this process involves a sequential transition from native pCRP through an activated pCRP toward mCRP (50, 51, 90). This transition can be triggered by interaction with LPC-enriched membranes or apoptotic cell surfaces in a Ca^2+^-dependent manner, with the conversion kinetics depending strongly on membrane composition and ligand availability (69). Under sustained membrane-associated conditions, substantial progression toward mCRP formation may occur over extended periods ranging from several hours to tens of hours. These timeframes, however, should not be regarded as fixed physiological rates, because CRP conversion kinetics are strongly influenced by membrane composition, Ca^2+^ availability, pH, oxidative status, and other features of the local microenvironment. Quantitative rate constants and a defined physiological half-time for the sequential pCRP-to-pCRP*-to-mCRP transition *in vivo* remain unavailable. Collectively, current evidence indicates that CRP conformational remodeling is limited under stable physiological conditions but can proceed over stimulus-dependent timescales ranging from minutes to several hours or longer in destabilizing and pathological microenvironments (50).

The isoform-dependent distribution of CRP is not confined to cardiovascular disease. Differential associations of pCRP and mCRP have also been described in autoimmune, infectious, neoplastic, and other acute inflammatory conditions (88). In systemic lupus erythematosus, circulating CRP isoforms have been linked to distinct clinical phenotypes and levels of disease activity, whereas mCRP has been detected both within tumor tissues and in the circulation of patients with colorectal cancer. In addition, conformationally altered CRP has been implicated in neutrophil activation and the formation of neutrophil extracellular traps during inflammatory responses (91). Together, these observations indicate that the biological consequences of CRP conformational remodeling extend well beyond vascular pathology and may represent a broader mechanism by which a circulating acute-phase protein acquires localized, context-dependent inflammatory functions. The distinct tissue distribution and biological activities of pCRP and mCRP therefore provide an important mechanistic link between CRP conformational conversion and its *in vivo* pathological and clinical relevance (92).

Importantly, evidence from both animal models and human-derived samples underscores the pathological relevance of CRP conformational conversion *in vivo*. In rat models of renal and striated-muscle ischemia/reperfusion injury, conformationally altered CRP accumulates at sites of tissue damage, where it promotes leukocyte recruitment and reactive oxygen species generation, directly implicating the pCRP-to-mCRP transition in the amplification of local inflammatory responses (80). Consistent with these experimental observations, mCRP has been identified within human atherosclerotic plaques and on circulating microparticles from patients with myocardial infarction (78), whereas native pCRP is predominantly confined to the soluble circulating compartment. Moreover, mCRP deposition in human atherosclerotic lesions is preferentially localized to inflamed and vulnerable plaque regions, indicating a close spatial association between CRP conformational conversion and vascular injury. Collectively, these findings suggest that pCRP and mCRP should be regarded not simply as structurally distinct forms of the same protein, but as functionally differentiated states whose localization and biological activities are shaped by the local inflammatory microenvironment (77, 93).

Nevertheless, the *in vivo* significance of mCRP remains incompletely established. Although multiple studies have detected conformationally altered CRP or mCRP-associated epitopes under pathological conditions, rigorous evidence directly demonstrating an essential functional role of mCRP *in vivo* is still limited. Among the more compelling observations, disease-associated exposure of mCRP-specific epitopes has been linked to clinical outcome, while evolutionary analyses further support the potential biological relevance of CRP conformational diversification. These findings provide important *in vivo* and evolutionary support, but do not yet establish mCRP as a definitively proven pathogenic effector *in vivo*.

### 4.3 Oxidative stress-mediated conformational remodeling and functional regulation

Neutrophils and macrophages activated at inflammatory sites produce large amounts of reactive oxygen species (ROS), establishing an oxidative microenvironment that contributes to the conformational regulation of CRP. Studies have shown that exposure of CRP to hydrogen peroxide (H_2_O_2_) (94), even in the absence of complete pentamer dissociation, can induce localized conformational destabilization and loosening. This oxidative modification resembles the effects of acidic pH and promotes the exposure of cryptic epitopes that are normally concealed within native pCRP. Notably, oxidative modification and the accompanying structural loosening of CRP have been reported to enhance its interaction with oxLDL. This observation further emphasizes that oxLDL-binding activity should not be regarded as an invariant property of native pCRP, but may emerge or increase as CRP undergoes conformational remodeling under oxidative conditions (95–97).

The surface-exposed methionine residues of CRP are primary targets for ROS-mediated oxidation (98). Upon oxidation to methionine sulfoxide, the hydrophilicity and steric properties of the methionine side chain undergo substantial alterations. This oxidative modification may affect CRP structure and function through two potential mechanisms: first, it may disrupt critical hydrophobic interactions and hydrogen-bond networks involved in maintaining the stability of the ligand-binding pocket or inter-subunit interfaces, thereby inducing local structural perturbations and functional alterations; second, the redox state of methionine residues may serve as a reversible post-translational regulatory mechanism (99, 100), dynamically modulating CRP activity, similar to redox-sensitive conformational regulation observed in certain signaling proteins (101).

Advanced biomolecular modeling provides a useful framework for evaluating how local molecular perturbations influence conformational stability and interaction networks. Molecular dynamics simulations, complemented by interaction-energy approaches such as Molecular Mechanics/Generalized Born Surface Area, have been widely applied to characterize the stability and residue-level interactions of biomolecular complexes (102). Because methionine oxidation converts a hydrophobic thioether side chain into a more polar sulfoxide form, such modifications may alter local packing, solvent exposure, and non-covalent interaction networks, thereby influencing conformational flexibility. In the context of CRP, analogous redox-state-specific simulations could be used to compare reduced and oxidized methionine-containing states and determine whether local oxidative modifications propagate toward ligand-binding regions or inter-subunit interfaces. Such approaches may provide a mechanistic link between experimentally observed ROS-induced structural loosening and the residue-level consequences of oxidative modification (94).

### 4.4 Thermal modulation of CRP conformational stability

Temperature variation represents another important physical factor influencing CRP structural dynamics and conformational stability (103). As a pentameric protein maintained by noncovalent interactions, CRP stability is governed by an integrated network of intramolecular hydrogen bonds, hydrophobic interactions, and inter-subunit contacts, all of which exhibit temperature dependence. Within the physiological temperature range, CRP maintains a stable pentameric architecture required for normal ligand recognition and functional regulation. However, deviations from physiological temperatures may increase molecular motions, enhance local structural flexibility, and alter the conformational equilibrium of CRP (104).

Elevated temperatures may perturb the hydrogen-bonding network and hydrophobic interactions that contribute to CRP quaternary structure stability, thereby weakening inter-subunit interfaces and promoting localized structural relaxation of the pentamer (92). Increased conformational flexibility may alter the exposure of specific surface regions, consequently affecting ligand-binding properties and interactions with immune-related molecules. Under sustained thermal stress, cumulative structural perturbations may further compromise pentamer integrity, favoring the transition of CRP toward more dynamic conformational states.

Furthermore, temperature variation rarely acts as an independent factor affecting CRP, but rather occurs together with other inflammatory microenvironmental changes, including acidification, oxidative stress, and membrane remodeling. Under such combined stimuli, elevated temperature may increase CRP conformational flexibility and reduce its structural tolerance to environmental perturbations, thereby shifting the conformational equilibrium toward activated states and increasing the likelihood of subsequent pCRP and mCRP formation (105). Therefore, temperature may serve as an important physical regulator of the CRP conformational landscape, contributing to the dynamic balance among structural stability, ligand recognition, and inflammatory functional states.

### 4.5 Chemical denaturant-induced perturbation of CRP Conformation

Chemical denaturants, such as urea and guanidine hydrochloride (GdnHCl), are commonly used to study the conformational stability and structural transition mechanisms of CRP by disrupting noncovalent interactions crucial for protein assembly (38). The stability of CRP’s pentameric structure, maintained by inter-subunit interactions, can be assessed through chemical denaturation. Studies have shown that treatment with GdnHCl or urea leads to structural changes in CRP, revealing hidden hydrophobic regions, altering secondary structures, and transitioning from pentameric to monomeric states (106). These chemically generated CRP conformations exhibit distinct structural and functional properties, influencing downstream effector recognition (107).

Urea- and GdnHCl-induced transitions provide valuable information regarding CRP structural stability, but these perturbations differ from biologically relevant regulatory mechanisms (108). While factors like Ca^2+^ depletion, acidic microenvironments, oxidative stress, or membrane-associated ligands regulate CRP activity through physiologically significant conformational change, chemical denaturants cause non-specific destabilization of protein structures by altering the energetic balance between folded and unfolded states. Therefore, urea and GdnHCl should be regarded as experimental probes for dissecting the conformational energy landscape of CRP rather than direct physiological inducers of pCRP-to-mCRP conversion (109).

To more precisely resolve intermediate states during urea- or GdnHCl-induced unfolding, chemical denaturation should be combined with orthogonal biophysical approaches that monitor different structural levels. Intrinsic tryptophan fluorescence and near-ultraviolet circular dichroism can detect early changes in tertiary packing, whereas far-ultraviolet circular dichroism reports alterations in secondary structure. Fluorescence-based hydrophobicity assays can reveal transient exposure of hydrophobic surfaces characteristic of partially unfolded or molten-globule-like states (38). Changes in oligomeric organization can be further resolved using size-exclusion chromatography coupled to multi-angle light scattering or analytical ultracentrifugation, allowing discrimination between intact pentamers, partially dissociated intermediates, and monomeric species (28). Hydrogen–deuterium exchange mass spectrometry could provide residue or peptide-level information on regions that become destabilized during unfolding, thereby identifying the structural segments and subunit interfaces involved in intermediate-state formation. Together, these complementary techniques would enable a more detailed reconstruction of the multistep unfolding pathway of CRP rather than treating denaturation as a simple two-state transition (110).

## 5 Conclusion and future perspectives

### 5.1 Summary of core mechanisms

The function of CRP relies primarily on maintaining of its native pentameric conformation through Ca^2+^-mediated stabilization and its ability to recognize specific ligands. The B-face of CRP contains a conserved Ca^2+^-dependent ligand-binding pocket, with each subunit harboring two cooperatively acting Ca^2+^-binding sites (Ca1 and Ca2). These sites are coordinated by residues like Asp60, Asn61, Glu138, Asp140, and main-chain carbonyl oxygens, collectively forming the Ca^2+^ coordination network. Asp140, among these residues, acts as a critical bridging residue contributing to the stabilization of the dual-calcium coordination system. Calcium ion binding maintains the local loop conformation, preserves the integrity of the ligand-binding pocket, and facilitates high-affinity recognition of ligands such as PC by CRP. Conversely, Ca^2+^ depletion destabilizes the binding pocket, leads to pentamer dissociation, and hampers ligand-binding capability.

Upon binding to pathogen- or damage-associated membrane ligands through its B-face, CRP may undergo conformational remodeling that alters the exposure and organization of effector-binding regions. This structural transition can modulate interactions with complement components, including C1q and CFH, indicating that CRP-mediated complement regulation is closely linked to its conformational state rather than ligand binding alone.

### 5.2 Outstanding challenges and future directions

The conformational dynamics of CRP within complex *in vivo* microenvironments are not fully understood. Further investigation is required to elucidate the environmental triggers, spatiotemporal dynamics, and regulatory mechanisms involved in governing the transition from pCRP to mCRP. To bridge the gap between *in vitro* structural biology and the complex *in vivo* microenvironment, future studies should incorporate conformation-sensitive live-cell imaging and Förster resonance energy transfer-based strategies. Such approaches would be particularly valuable for validating membrane-associated conformational transitions proposed from *in vitro* studies, including the formation of activated pCRP intermediates preceding mCRP generation. Site-specific labeling strategies targeting defined positions within neighboring CRP subunits could enable real-time monitoring of changes in inter-subunit proximity during the pCRP-to-pCRP* transition, while Fluorescence lifetime imaging microscopy–Förster resonance energy transfer may provide more robust quantitative readouts in heterogeneous cellular environments. In parallel, fluorescently labeled CRP combined with conformation-selective antibodies or nanobodies could be used for live-cell or intravital imaging to track CRP recruitment and conformational conversion at inflammatory sites. Coupling these approaches with pH, Ca^2+^, or ROS-sensitive reporters may further link CRP structural transitions to disease-associated microenvironmental changes. Understanding the assembly, dissociation, and biological functions execution of different CRP conformational states on cellular membranes is a crucial area for future research. Moreover, while CRP can transiently from complexes with C1q, C3b, Fcγ receptors, and other plasma proteins, high-resolution structural data for these complexesare scarce. Determining the structures of CRP complexes with downstream effector molecules under physiological and disease-associated conditions will provide deeper insights into the molecular mechanisms that underlie CRP-mediated complement activation and phagocytic regulation.

Insufficient understanding persists regarding the variations in CRP structural stability across species and their evolutionary implications. Moreover, further research is warranted to ascertain if CRP gene polymorphisms impact individual susceptibility to infection, inflammation, and chronic diseases by modifying protein conformation and function. Despite the clinical potential demonstrated by CRP-targeted inhibitors or stabilizers developed through structure-based approaches, challenges persist in their translation, including issues with target specificity, pharmacokinetics, and safety profiles. In the future, CRP research should advance by integrating sophisticated methods such as cryo-electron microscopy, X-ray crystallography, molecular dynamics simulations, and live-cell imaging. This integration is crucial for systematically characterizing CRP conformational dynamics across diverse microenvironments, with a particular focus on elucidating the molecular mechanisms that govern the transition from pCRP to mCRP in environments characterized by reduced calcium availability, acidic stress, oxidative stress, and membrane association.

Intervention strategies targeting CRP can involve stabilizing the pCRP pentameric structure, modulating Ca^2+^-dependent ligand recognition, preventing the conformational transition from pCRP to mCRP, and disrupting interactions between CRP and downstream effector molecules such as C1q. Therapeutic approaches focusing on CRP conformational dynamics and functional regulation could offer novel opportunities for the precise management of diseases linked to inflammation, owing to the ongoing progress in structural biology and drug discovery technologies. Future research should prioritize the development of specific drugs targeting CRP and small-molecule inhibitors that can influence CRP conformational changes, stabilize pCRP, hinder the transition from pathogenic pCRP-to-mCRP, or disrupt CRP-effector interactions. Among currently characterized sites, the Ca^2+^-dependent phosphocholine/phosphoethanolamine-binding pocket on the B-face has the strongest experimental support as a pharmacological target, as phosphocholine-mimetic ligands can stabilize native CRP and interfere with membrane-induced conformational conversion. Beyond the ligand-binding pocket, the inter-subunit interfaces represent a particularly attractive allosteric region because they directly control subunit packing and pentamer integrity, whereas central-pore-associated clefts remain more speculative candidates requiring further structural validation. However, their relative druggability and potential for allosteric stabilization have not yet been systematically evaluated. Progress in structural biology and drug discovery techniques could aid in developing specific CRP modulators, creating novel prospects for targeted anti-inflammatory treatments.

A major challenge in translating CRP conformational biology into clinical practice is the reliable discrimination and quantification of individual CRP isoforms in soluble biological samples. Routine clinical assays primarily measure total circulating CRP and generally do not differentiate between pCRP and mCRP, despite their distinct structural and biological properties. This limitation is particularly important because pCRP represents the predominant soluble form in the circulation, whereas mCRP is preferentially associated with membranes, microparticles, extracellular vesicles, and inflamed tissues. Several studies have attempted isoform-specific detection using customized or in-house immunoassays, including Enzyme-linked immunosorbent assay-based approaches employing antibodies against conformationally exposed epitopes. However, variability in antibody specificity, sample processing, calibration standards, and the potential for CRP interconversion or aggregation during sample handling continues to hinder cross-study comparability and assay standardization. Future efforts should therefore prioritize the development and rigorous validation of standardized, isoform-specific assays capable of reliably quantifying pCRP and mCRP in serum or plasma. Such methodological advances will be critical for establishing whether CRP isoform profiling offers diagnostic or prognostic information beyond that provided by conventional measurements of total CRP.
